# Pharmacological activation of lysophosphatidic acid receptors regulates erythropoiesis

**DOI:** 10.1038/srep27050

**Published:** 2016-05-31

**Authors:** Kuan-Hung Lin, Ya-Hsuan Ho, Jui-Chung Chiang, Meng-Wei Li, Shi-Hung Lin, Wei-Min Chen, Chi-Ling Chiang, Yu-Nung Lin, Ya-Jan Yang, Chiung-Nien Chen, Jenher Lu, Chang-Jen Huang, Gabor Tigyi, Chao-Ling Yao, Hsinyu Lee

**Affiliations:** 1 Department of Life Science, National Taiwan University, Taipei, Taiwan; 2 School of Biomedical Science, Ohio State University, Columbus, OH, USA; 3 Graduate Institute of Oral Biology, School of Dentistry, National Taiwan University, Taipei, Taiwan; 4 Department of Surgery, National Taiwan University Hospital and College of Medicine, Taipei, Taiwan; 5 Department of Pediatrics, Taipei Veterans General Hospital, Taipei, Taiwan; School of Medicine, National Yang-Ming University, Taipei, Taiwan; 6 Department of Electrical Engineering, National Taiwan University, Taipei, Taiwan; 7 Department of Physiology, University of Tennessee Health Science Center, Memphis, TN, USA; 8 Department of Chemical Engineering and Materials Science, Yuan Ze University, Taoyuan, Taiwan; 9 Angiogenesis Research Center, National Taiwan University, Taipei, Taiwan; 10 Research Center for Developmental Biology and Regenerative Medicine, National Taiwan University, Taipei, Taiwan; 11 Center for Biotechnology, National Taiwan University, Taipei, Taiwan; 12 Institute of Biological Chemistry, Academia Sinica, Taipei, Taiwan

## Abstract

Lysophosphatidic acid (LPA), a growth factor-like phospholipid, regulates numerous physiological functions, including cell proliferation and differentiation. In a previous study, we have demonstrated that LPA activates erythropoiesis by activating the LPA 3 receptor subtype (LPA_3_) under erythropoietin (EPO) induction. In the present study, we applied a pharmacological approach to further elucidate the functions of LPA receptors during red blood cell (RBC) differentiation. In K562 human erythroleukemia cells, knockdown of LPA_2_ enhanced erythropoiesis, whereas knockdown of LPA_3_ inhibited RBC differentiation. In CD34^+^ human hematopoietic stem cells (hHSC) and K526 cells, the LPA_3_ agonist 1-oleoyl-2-methyl-sn-glycero-3-phosphothionate (2S-OMPT) promoted erythropoiesis, whereas the LPA_2_ agonist dodecyl monophosphate (DMP) and the nonlipid specific agonist GRI977143 (GRI) suppressed this process. In zebrafish embryos, hemoglobin expression was significantly increased by 2S-OMPT treatment but was inhibited by GRI. Furthermore, GRI treatment decreased, whereas 2S-OMPT treatment increased RBC counts and amount of hemoglobin level in adult BALB/c mice. These results indicate that LPA_2_ and LPA_3_ play opposing roles during RBC differentiation. The pharmacological activation of LPA receptor subtypes represent a novel strategies for augmenting or inhibiting erythropoiesis.

Lysophosphatidic acid (LPA) is generated by the lysophospholipase D enzyme autotaxin (ATX) and present in biological fluids at concentrations that can reach micromolar levels in serum. At least six G-protein-coupled LPA receptors (LPARs) have been described. The LPA_1_, LPA_2_, and LPA_3_ GPCR are belong to the endothelial differentiation gene family^1^, whereas LPA_4_, LPA_5_, and LPA_6_ belong to in the purinergic receptor cluster^3^. It has been shown that LPARs are involved in stem cell differentiation^4^. Previous results have indicated that LPA induces embryonic stem cell proliferation and differentiation by activating the phospholipase C (PLC)/Ca^2+^ signaling axis^6^. LPARs have also been identified in murine and human pluripotent stem cells, including in hematopoietic and embryonic stem cells^7^. Furthermore, it has been reported that induced pluripotent stem cells (iPSC) express LPA_1−4_ GPCR and that LPA induces iPSC differentiation and proliferation by activating the Rho/ROCK pathway^8^. However, the role of LPA and its GPCR during hematopoiesis remain elusive.

Hematopoiesis occurs in two waves during vertebrate development, a short-lived “primitive wave” that is characterized by embryonic globin expression; and a later “definitive wave” that develops intraembryonically in the aorto-gonadal-mesonephros region of the embryo^9^,^10^,^11^. The primitive wave of hematopoiesis is a transient process during embryonic development. This process produces unipotent blood cells that act as oxygen transporters, and it is therefore essential for the viability of the embryo^12^. The definitive wave of hematopoiesis occurs in the fetal liver to produce hematopoietic stem cells (HSCs) that support the differentiation of all blood cell lineages^13^,^14^. Recent studies suggest that LPA plays an important role in regulating primitive hematopoiesis through activation of LPA_1_^15^. It has also been shown that LPA promotes myeloid differentiation in the human bone marrow microenvironment^16^. LPA was recently shown to promote the differentiation of the myeloid/macrophage lineage from human CD34^+^ hematopoietic progenitors via activation of LPA_2_ *in vitro*^4^. Thus, corollary evidence suggests that LPA might regulate HSC differentiation and blood cell homeostasis.

We have demonstrated previously the role of LPA_3_ in red blood cell (RBC) differentiation^17^. In the present study, we report the pharmacological dissection of specific roles of LPA GPCR subtypes in three different species. Our results demonstrate that LPA_2_ and LPA_3_ exert opposing roles on RBC differentiation *in vitro* and *in vivo*. We also highlighted that 2S-OMPT, a LPA_3_ agonist, is a potential drug candidate that enhances erythropoiesis *in vivo*.

## Results

### LPA_2_ and LPA_3_ expression is differentially regulated during hemin-induced erythropoiesis in K562 cells

Using human hematopoietic stem cells we have demonstrated previously that LPA enhances erythropoiesis by activating LPA_3_^17^. To examine the expression patterns of LPAR during differentiation, we applied hemin-induced erythropoiesis model system using the K562 cell line^18^. We first evaluated mRNA expression of lpar1, lpar2, and lpar3. Under unstimulated conditions, K562 cells abundantly expressed lpar2, a moderate level lpar3; however, lpar1 was barely detectable (Supplemental Fig. 1a). Upon hemin induction, erythropoiesis took place indicated by upregulation of γ-globin mRNA^19^ (Supplemental Fig. 1b). During differentiation, mRNA expression levels of lpar2 decreased significantly (p < 0.05) after 48 h of culture (Supplemental Fig. 1c). In contrast, lpar3 mRNA expression level remained unchanged (Supplemental Fig. 1d).

### Opposing regulation of erythropoiesis by LPA_2_ and LPA_3_

**Figure 1:**
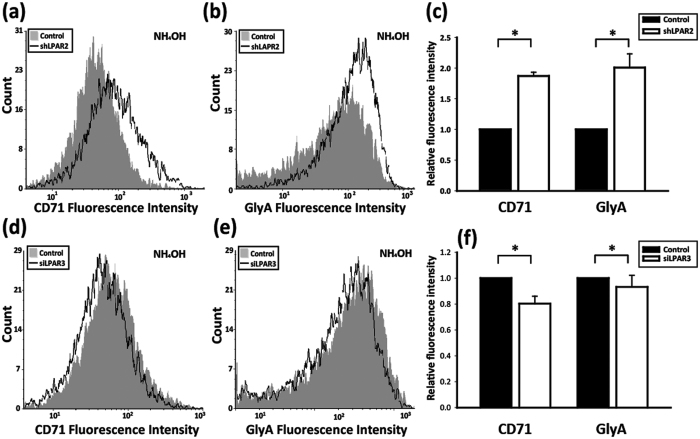
The role of LPA GPCR in RBC differentiation of the K562 cell line. (**a**–**c**) Knockdown of LPAR_2_ using lentiviral shRNA and (**d**–**f**) knockdown of LPAR_3_ by siRNA. CD71-FITC-conjugated and GlyA-PE-conjugated antibodies were used to stain and analyze for the expression of CD71 and GlyA. The geometric mean of fluorescence was used to quantify the results. The quantitative data presented as the mean ± SD of at least three independent experiments. *p < 0.05 and **p < 0.01 indicate significant differences compared with vehicle control.

The decrease of lpar2 expression during differentiation suggests its role in the regulation of erythropoiesis. We hypothesized that downregulation of LPA_2_ might represent disinhibition signal and promotes the erythroid differentiation of K562 from the undifferentiated status. Using lentiviral transduction, we selected K562 cells that stably express LPAR2 shRNA (shLPAR2) with 80% knockdown efficiency compared to scramble controls (Control) (Supplemental data 1e). Flow cytometry showed that shLPAR2 expressing cells displayed higher expression of the erythroid markers CD71 and glycophorin A (GlyA) compared to controls (Fig. 1a–c and Supplemental Fig. 2a–c), indicating that knockdown of LPA_2_ promoted erythropoietic differentiation of K562 cells. Based on our previous observation in HSC demonstrating that knockdown of LPA_3_ suppressed erythropoiesis^17^, we transfected LPA_3_ siRNA (siLPAR3) into K562 cells and found downregulation of the erythropoietic markers CD71 and GlyA (Fig. 1d–f and Supplemental Fig. 2d–f). These results taken together indicate that knockdown of LPA_2_ and LPA_3_ exert opposing effects on erythropoiesis in the K562 model.

### Pharmacological manipulation of LPAR of hemin-induced erythropoiesis in K562 cells

**Figure 2:**
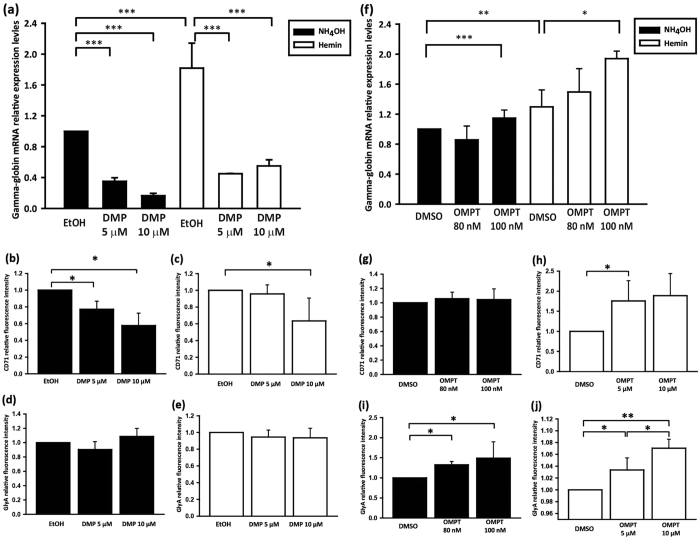
Pharmacological treatment with different LPA receptor agonists and antagonists regulates erythropoiesis in K562 cells. K562 cells were treated, either in the presence (indicated as black bars) or absence of hemin (indicated as white bars), plus (**a**) LPA_2_ agonist DMP at 5 μM and 10 μM or (**f**) LPA_3_ agonist 2S-OMPT at 80 nM and 100 nM. The mRNA expression levels of γ-globin were analyzed by qPCR. K562 cells were treated with (**b**–**e**) LPA_2_ agonist DMP at 5 μM and 10 μM and (**g**–**j**) LPA_3_ agonist 2S-OMPT at 80 nM and 100 nM. Fluorescence intensity of CD71 (**b**,**c**,**g**,**h**) and GlyA (**d**,**e**,**i**,**j**) staining was quantified by Flow cytometry. Data are represent the mean ± SD of three independent experiments. *p < 0.05, **p < 0.01, and ***p < 0.005 indicate significant differences compared with vehicle control.

Because LPA_2_ and LPA_3_ appeared to play opposing roles in the regulation of erythroid differentiation, we explored the use of pharmacological manipulation specifically targeting LPA_2_ and LPA_3_ receptors to confirm our finding from the knockdown experiments. We stimulated K562 cells with the LPA_2_-selective agonist, DMP and found that it significantly decreased the expression of γ-globin mRNA (Fig. 2a). The erythroid differentiation marker CD71 was also downregulated by DMP treatment (Fig. 2b,c). However, the expression of GlyA was unaffected (Fig. 2d,e). These results are consistent with the hypothesis that the activation of LPA_2_ inhibits erythroid differentiation. We also found that erythropoiesis was enhanced by treating K562 cells with the LPA_3_-specific agonist, 2S-OMPT. We found that exposure to as low as 80-to-100 nM 2S-OMPT resulted in significant upregulation of γ-globin expression (Fig. 2f). Flow cytometry analysis showed that 2S-OMPT increased GlyA protein expressions in a dose-dependent manner (Fig. 2i,j). Furthermore, we found that CD71 was induced by combined treatment with 2S-OMPT and hemin (Fig. 2h) whereas, it was unaffected in the absence of hemin (Fig. 2g). Considering that LPA_1_ expression in K562, is almost undetectable, these finding might suggest that activation of LPA_3_ but not LPA_1_, enhances erythroid differentiation. The mRNA expression profiling of LPA_2_ and LPA_3_ was also evaluated following LPAR agonist treatment to exclude transcriptional alterations in LPAR expression due to ligand exposure. The LPA_2_ specific agonist GRI977143 (Supplemental Fig. 3a,b) and the LPA_3_ agonist 2S-OMPT (Supplemental Fig. 3e,f) caused no detectable alteration in the expression patterns of LPA_2_ and LPA_3_ mRNA. However, the LPA_2_ agonist DMP significantly inhibited LPA_2_ mRNA expression (Supplemental Fig. 3c,d). The mechanism of decreased LPA_2_ mRNA expression remains unclear. Nonetheless, these results confirm the opposing regulation of erythropoiesis by LPA_2_ and LPA_3_ in K562 cell line model.

### Opposing effects of LPA_2_ and LPA_3_ in erythropoiesis of CD34^+^ hHSC

**Figure 3:**
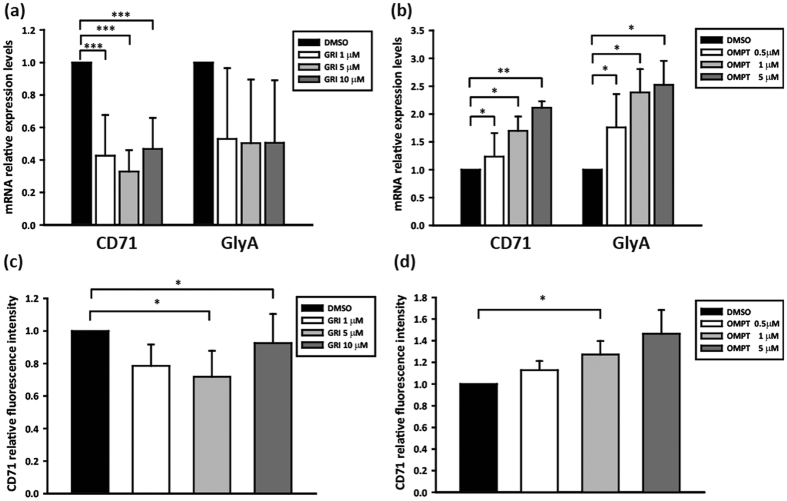
Pharmacological treatment with LPA_2_ and LPA_3_ agonists of CD34^+^ hHSC. Umbilical blood-derived CD34^+^ hHSC were cultured with GRI and 2S-OMPT separately for six days under EPO induction. (**a**,**b**) The cells were harvested and the expression of CD71 and GlyA RNA was analyzed using qPCR. (**c**,**d**) The CD71 protein expression was analyzed by flow cytometry. Data are the mean ± SD of at least three independent experiments. *p < 0.05, *p < 0.01, and ***p < 0.005 indicate significant differences compared with vehicle control.

To further consolidate our hypothesis, we isolated CD34^+^ hHSC from umbilical cord blood^20^ and treated the cells with the LPA_2_ agonist GRI. In CD34^+^ hHSC, GRI inhibited CD71 mRNA expression during erythropoiesis elicited by erythropoietin and stem cell factor treatment. However, activation of LPA_2_ had no significant effect on GlyA mRNA expression (Fig. 3a). This might be due to the fact that CD71 is an early RBC marker as opposed to GlyA, which is a late RBC marker^21^. In contrast, the LPA_3_ agonist 2S-OMPT significantly enhanced the mRNA expression level of CD71 and GlyA (Fig. 3b). Furthermore, we also examined CD71 protein levels by flow cytometry and found that it paralleled changes in the mRNA expression pattern: GRI decreased, whereas 2S-OMPT increased CD71 protein expression, (Fig. 3c,d). These results are consistent with our findings in the K562 model, corroborating that LPA_2_ inhibits, whereas activation LPA_3_ promotes erythropoiesis in hHSC.

### Pharmacological blockade of LPA signaling inhibits hematopoiesis in zebrafish

**Figure 4:**
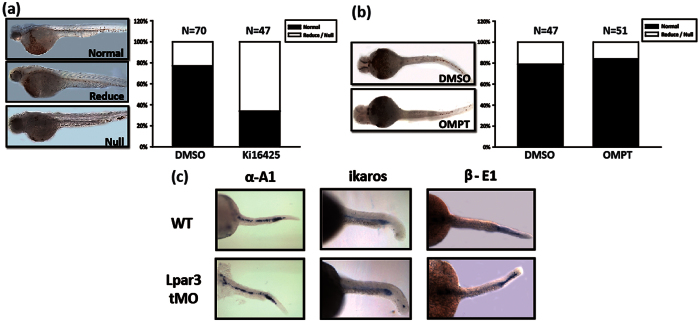
Characterization of the function of LPA_3_ during primitive erythropoiesis in zebrafish embryos. Zebrafish embryos were immersed in (**a**) the LPA_1/3_ antagonist Ki16425 or (**b**) the LPA_3_ agonist 2S-OMPT for 48 h. O-dianisidine staining was classified as normal, moderate, or severe based on the expression level of embryonic blood in the CHT region. The right-hand panel shows the quantitative results. (**c**) Whole-mount *in situ* hybridization showed the mRNA expression of α-A1, β-E1 globin and ikaros in zLPAR_3_ tMO-injected or wild type embryos.

Our *in vitro* models have established that pharmacological activation of LPA_3_ enhances, whereas LPA_2_ suppresses erythroid differentiation. We evaluated these effects using the zebrafish model *in vivo*^17^. In zebrafish, the constitutive expression of LPA_3_ at all developmental stages suggests that it plays an important role during early embryogenesis^22^. To investigate whether LPA_3_ regulates erythropoiesis, we exposed zebrafish embryos to the LPA_1_/LPA_3_ mixed antagonist Ki16425. Expression of hemoglobin in zebrafish embryos were detected by o-dianisidine staining after 24 h of treatment with Ki16425. Ki16425 treatment resulted in moderate-to-severe phenotypes in 60% of the fish (Fig. 4a) suggesting that blockade of LPA_1_ and LPA_3_ reduced hemoglobin production in zebrafish embryos. However, no significant effect was observed in response to 2S-OMPT treatment (Fig. 4b). To further evaluate the importance of LPA_3_ signaling during zebrafish erythropoiesis, we blocked zLPAR_3_ by injecting 8 ng of morpholino (MO) zLPAR3, which targets the non-overlapping site near the translation initiation site. The WISH results showed no perturbation of the of primitive erythrocyte marker hbaa1 (α-A1 globin)^23^, myeloid marker hbbe1.1 (β-E1 globin)^23^ and ikzf1 (ikaros)^24^, which is a marker expressed both in primitive and definitive erythrocytes (Fig. 4c). These results indicate that the effect of LPA_3_ on erythropoiesis is not dominant at 48 hpf, which represents the primitive hematopoiesis stage.

### Blockade of LPA_3_ perturbs erythroid differentiation during the definitive wave of hematopoiesis in zebrafish

**Figure 5:**
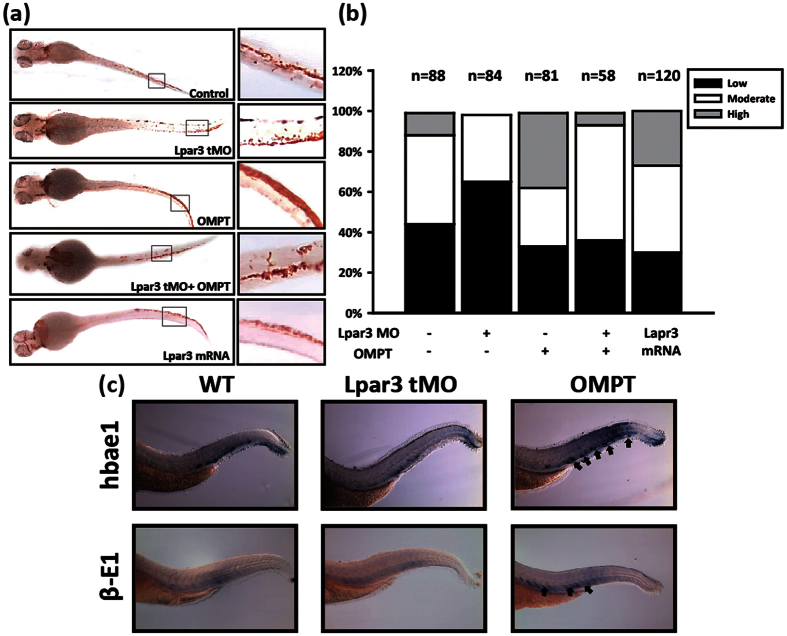
Analysis of the roles of LPA_3_ during definitive erythropoiesis in zebrafish. (**a**) O-dianisidine staining showed the effects of LPAR_3_ mRNA, 2S-OMPT, and LPAR_3_-MO during definitive erythropoiesis at 96 hpf in the CHT region. (**b**) The quantitative results for (**a**). (**c**) A whole-mount *in situ* hybridization showing the mRNA expression levels of α-E1 and β-E1 in the wild-type, LPAR_3_-MO, and 2S-OMPT treatment groups.

The primitive wave mainly produces erythrocytes in the intermediate cell mass at 24 hpf, whereas the mature erythroids that differentiate in the definitive wave after 48 hpf^11^,^2^5 are found in caudal hematopoietic tissue (CHT)^26^,^27^,^28^. Definitive erythrocytes are enriched in the posterior blood island from 3.5 days post-fertilization (dpf)^29^; hence, we selected 96 hpf as the sampling time point. Hemoglobin expression increased after injection with zLPAR3 mRNA or treatment with 2S-OMPT for 96 h. Exposure to 2S-OMPT also rescued the erythropoietic defect caused by zLPAR_3_ tMO injection (Fig. 5a,b). The expression of the definitive erythroid lineage markers hemoglobin genes, including hbae1 (α-E1 globin) and β-E1 globin, was increased (Fig. 5c). These results demonstrate that LPA_3_ is an important regulator of the definitive wave of erythropoietic differentiation in HSC.

### Knockdown of LPA_2_ increases hemoglobin expression during definitive hematopoiesis in zebrafish

**Figure 6:**
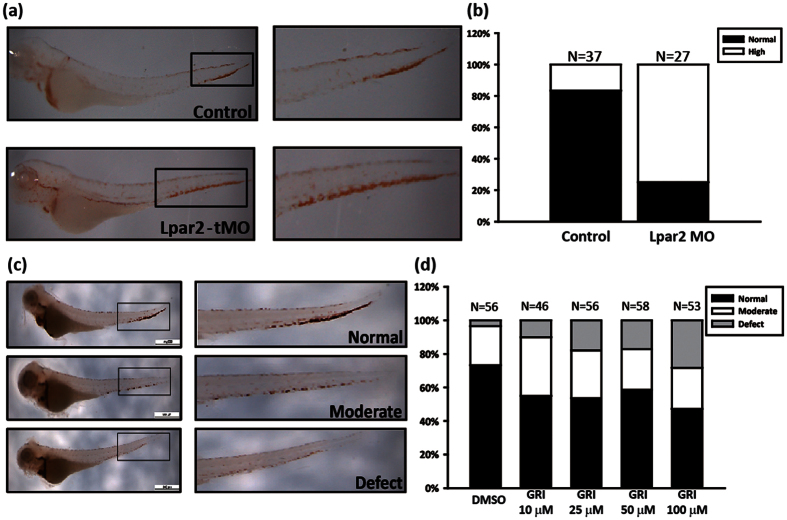
Characterization of the effects of LPA_2_ during definitive erythropoiesis. The effects of LPAR_2_ MO revealed defective erythropoiesis in the CHT region based on o-dianisidine staining. (**b**) The quantitative results for (**a**). (**c**) The effects of the LPA_2_ agonist GRI after treatment for 96 h. O-dianisidine staining levels were classified as normal, moderate, or defective based on the expression levels in the embryonic blood. (**d**) The quantification hemoglobin expression after treatment with different concentrations of GRI for four days.

We also investigated the roles of LPA_2_ during erythropoiesis in zebrafish by injecting zLPAR2 tMO into the yolk of embryos at the one-cell stage. Non-injected and MO-injected embryos were collected at 4 dpf and subjected to o-dianisidine staining. Knockdown of zLPAR2 using MOs enhanced the hemoglobin levels in CHT (Fig. 6a,b). To confirm the results obtained with the MO-injected embryos, the embryos were treated with the LPA_2_ agonist GRI. These results indicated that the percentage of embryos with severe RBC defect increased with the concentration of GRI (Fig. 6c,d). Taken together, our findings indicate that LPA_2_ plays an inhibitory role in RBC development in zebrafish.

### Activation of LPA receptors regulates erythropoiesis in mice

**Figure 7:**
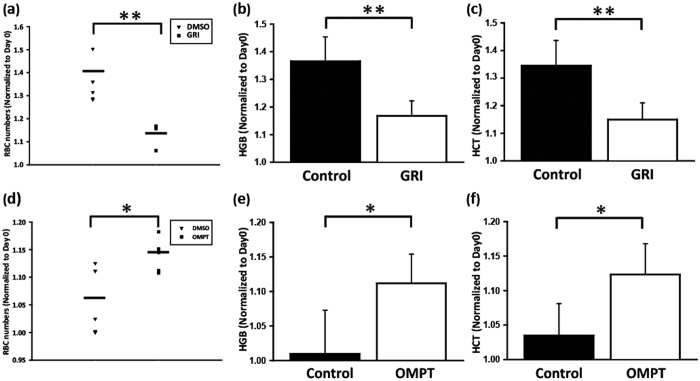
Effects of LPA2 and LPA_3_ on erythropoiesis in mice. BALB/C mice were injected peritoneally with (**a**–**c**) 0.5 mg/kg 2S-OMPT and (**d**,**e**) 1 mg/kg GRI every day. The mice were sacrificed on post-injection day eight. Blood samples were isolated from the facial vein. (**a**,**d**) the RBC counts, (**b**,**e**) HGB and (**c**,**f**) HCT were determined. Data are normalized to the results obtained from day 0. The quantitative data represent the mean ± SD of at least four mice. *p < 0.05, *p < 0.01, and ***p < 0.005 indicate significant differences compared with vehicle control.

To further confirm the relationship between LPA_2_ and LPA_3_ GPCR in erythropoiesis of mammals, we injected 2S-OMPT and GRI intraperitonealy into 3-week-old BALB/c mice. The animals were sacrificed in post-injection day seven and blood samples were collected. The RBC count, HGB, and HCT decreased significantly after GRI injection (Fig. 7a–c), and conversely increased under 2S-OMPT treatment (Fig. 7d–f). These results indicate that activation of LPA_3_ promotes whereas activation of LPA_2_ inhibits RBC differentiation in mice.

## Discussion

Recent studies have reported the importance of LPA during mesenchymal stem cell differentiation^30^ and in the maintenance of multipotency of neuronal stem cells^31^. However, little is known about effect of LPA in hematopoiesis. Our previous study demonstrated that in the presence of EPO, LPA is an important regulator of erythropoiesis via LPA_3_ activation during EPO induction^17^. We also demonstrated the opposing roles of LPA_2_ and LPA_3_ during megakaryocytic differentiation in the K562 cell line^32^. Herein, using *in vivo* and *in vitro* models, we confirmed the role of LPA_3_ and the involvement of LPA_2_ in RBC differentiation. We also demonstrated for the first time that erythropoiesis can be modulated by treatment with specific agonists of LPARs.

2S-OMPT, a selective LPA_3_ agonist, has already been tested on renal function^33^, neurite growth^34^, and smooth muscle contraction^35^. We now have demonstrated that activation of LPA_3_ by 2S-OMPT also enhances hemoglobin mRNA transcripts, expression of erythroid markers, and the number of circulating RBC. Interestingly, 2S-OMPT treatment appeared to be more effective on GlyA expression than on CD71 in K562 cells and hHSC. During early erythrocytic development, CD71 is upregulated in erythroblasts, whereas GlyA increases subsequently during the terminal differentiation stage^21^. The differential responses of 2S-OMPT on GlyA and CD71 expression suggests LPA_3_ exerts its effect on late erythropoiesis. Using the zebrafish model, hemoglobin levels were decreased within 24 h of Ki16425 treatments. However, activation of LPA_3_ by 2S-OMPT enhanced hemoglobin expression only during the late stage of embryonic development. Consistent with these observations, LPA_1_ has been reported as a developmental cue to regulate early erythropoiesis^15^. These results suggest that LPA_1_ and LPA_3_ regulate erythropoiesis during different stages of development. Based on our findings here we propose that LPA_3_ is primarily involved during definitive erythropoiesis and is responsible for terminal erythrocyte differentiation. Traditionally, administration of recombinant human EPO (rHuEPO) is the most common treatment for anemia. However, numerous side effects of EPO have been reported^36^. In cancer-related anemia, activation of EPO receptor might also promote cancer progression and metastasis^37^,^38^,^39^. Evidence exists that rHuEPO-induced erythropoiesis also has side effects on the sensitivity to radiotherapeutic or chemotherapeutic outcomes in cancer patients^40^. Our results demonstrated that 2S-OMPT-treated mice had more RBC, higher HGB expression, and HCT ratio, suggesting a novel potential therapeutic strategy for the treatment of erythropoietic disorders. Appropriate administration of 2S-OMPT or similar specific LPA_3_ agonists might lower EPO dose necessary, thereby reducing the risk of undesirable side effects of long-term EPO therapy.

In the present study, we reported significant downregulation of LPA_2_ transcripts during hemin-induced erythropoiesis. In contrast to 2S-OMPT, activation of LPA_2_ by the DMP or GRI agonists suppressed erythroid marker gene expression. Knockdown of LPA_2_ promoted erythrocyte differentiation both *in vitro* and *in vivo*, suggesting that downregulation of LPA_2_ may provide a signal for erythroid-lineage commitment. Paradoxically, CD71 was more responsive to the activation of LPA_2_ in both the K562 and hHSC models. We propose that LPA_2_ exerts an inhibitory control at the early megakaryocyte-erythroid progenitor (MEP) stage by blocking terminal erythroid differentiation. Indeed, our previous data showed that activation of LPA_2_ inhibits the megakaryocytic transcriptional programs^32^. The high expression levels of LPA_2_ in unstimulated K562 also suggest its role in the maintenance of undifferentiated status. We propose that LPA_2_ signaling might be a molecular switch, which determines early HSC cell fate. It was reported that activation of LPA_2_ by GRI mitigates hematopoietic radiation syndrome^4^, suggesting activation of LPA_2_ acts as a radioprotector for HSC self-renewal. Given that GRI treatment significantly reduces circulatory RBC numbers, our results suggest the potential application of LPA_2_ agonists on several myeloproliferative diseases, including thrombocythemia, polycythemia vera, and primary myelofibrosis^41^.

Within the hematopoietic niche, several microenvironmental factors are necessary for HSC development^42^. Furthermore, interaction of cell surface molecules between bone marrow stromal cell and HSC also play an important role during hematopoiesis^43^. Previous studies reported that LPA promotes the expression of many of these factors during hematopoiesis^44^,^45^ in endothelial cells in the hematopoietic niche. Recent reports also demonstrated the role of LPA during early hematopoiesis^15^. In addition, the major catalytic enzyme of LPA, autotoxin, is highly expressed in the vicinity of the myeloid progenitors^16^. Therefore, LPA might be presented as a constitutive signal during myeloid differentiation. Our results demonstrated that activation of LPA_2_ and LPA_3_ exert opposing effects on erythropoiesis. We propose that decision of MEP cell fate might depend on the temporal pattern expression of these two LPA GPCR subtypes. Nonetheless, the downstream mechanisms originating from LPA_2_ and LPA_3_ that regulate erythropoiesis/megakaryopoiesis require further investigation.

Overall, our results suggest that agents similar to GRI and 2S-OMPT could become powerful drug candidates to regulate erythropoiesis. Additionally, we have also demonstrated the inhibitory role of LPA_2_ during RBC differentiation. These observations combined may facilitate the development of new treatment strategies for erythropoietic disorders.

## Methods

### Cell culture

K562 cells were cultured in RPMI supplemented with 10% FBS as described previously^32^. Mononuclear cells were isolated from umbilical cord blood by Ficoll-Paque (Amersham Biosciences, Uppsala, Sweden) density gradient centrifugation after obtaining the donor’s consent according to governmental regulations (“Guidelines for collection and use of human specimens for research”, Ministry of Health and Welfare, Taiwan) approved by the Institutional Review Board of the Taoyuan General Hospital, Taiwan. The isolation and culture methods for hHSC were described previously^17^. Briefly, human CD34^+^ hHSC were isolated from mononuclear cells by magnetic micro-bead isolating methods using Direct Progenitor Isolation Beads (Miltenyi Biotech, Bergisch Gladbach, Germany) and MACS LS-columns (Miltenyi Biotech). 2.5 × 10^5^ hHSC cells were cultured in 5 ml of EDM (5 × 10^4 ^ cells/ml) in the presence of SCF (50 ng/ml) and EPO (6 IU/ml) for 6 days.

### Pharmacological treatment

A 1 mM stock solution of LPA (18:1, Sigma-Aldrich), 2S-OMPT (Cayman Chemical), Ki16425 (Cayman), GRI977143^46^, dodecyl monophosphate (DMP; Sigma-Aldrich) and sphingosine-1-phosphate (Sigma-Aldrich) was prepared respectively as described in our previous report^32^. K562 cells were serum-starved for 12–16 h and cultured in 0.5% FBS medium containing the agonist and antagonist in the presence of hemin for 48 h. CD34^+^ hHSC were cultured with the test compounds diluted in the medium with 0.005% fatty acid-free bovine serum albumin.

### RNA isolation and real-time polymerase chain reaction (RT-PCR)

Total RNA was isolated using the TRIzol^®^ reagent (Thermo-Fisher). Thirty zebrafish embryos were ground up and total RNA was collected from the whole embryos. The level of each target mRNA was evaluated using quantitative RT-PCR (qPCR) based on the real-time threshold cycle and normalized against the amount of GAPDH. The specific primer sequences were as follows: *lpar1*: forward (5′->3′): TTCAACTCTGCCATGAA CCCC, reverse (3′->5′): CTAAACCAC AGAGTGGTCATT; *lpar2*: forward (5′->3′): ACACTT CTGGCACTGC CTCT, reverse (3′->5′): AGGCTGAGTGTGGTCTCTCG; *lpar3*: forward (5′->3′): TCAGC AGGAGTGACACAGGCAG, reverse (3′->5′): GGAAGTGCTTTTATTGCAGACT; *γ-globin*: forward (5′->3′): GCAGCTTGTCA CAGTGCAGTTC, reverse (3′-> 5′): TGGCAAGAAGGTGCTGACTTC; *CD71*: forward (5′>3′): GGATAAAGCGGTTCTTGGTACC, (3′-> 5′): CCAGTAACCGGA TGCTTCACA; *GlyA*: forward (5′->3′): ACAGACAAATGATACGCACAAACGGG, reverse (3′->5′): GGGCTTTTCTTTAT CAGTCGGCGA; *GAPDH*: forward (5′->3′): AAGGTGAAGGTCGGAGTC, reverse (3′->5′): TGTAGTTGAGGTCAATGAAGG.

### Flow cytometry

K562 cells and CD34^+^ hHSC were collected in phosphate-buffered saline (PBS) and stained with anti-CD71-FITC antibodies and anti-GlyA-PE antibodies (BD Bioscience Pharmingen; San Diego, CA, USA) for 30 min. Mouse bone marrow cells were collected in PBS and stained with anti-CD71-FITC antibodies (BD Bioscience Pharmingen) for 30 min. The stained cells were then washed three times and resuspended in PBS. All the cell samples were analyzed using a Cyto-flow instrument (Partec; Muenster, Germany) and the FCS Express software (De Novo; Los Angeles, CA, USA).

### RNAi transfection

Production of lentiviral stocks was described in detail in our previous study^47^. The LPA_2_ shRNA template was inserted into the pLKO.1 lentiviral vector = purchased from the National RNAi Core Facility Platform, Academia Sinica. The shRNA target sequence was: 5′-CCTGGTCAAGACTGTTGTCAT-3′ (shLPAR2; TRCN0000221131). The transfection and selection methods for transient or stable knockdown cells were described in our previous study^32^.

### Maintenance of zebrafish

The zebrafish were maintained according to an approved National Taiwan University Zebrafish Core Laboratory standard operating protocol for animal use. The zebrafish handling, breeding, and staging methods were performed as described previously^17^,^48^.

### Morpholino oligonucleotides

The MO stocks were prepared in sterile double-distilled water and 4-ng MO was injected with 5% phenol red. MO sequences were as follows: MO-*zLpar2*: 5′-CCAGCCCTAAAACACAGGAAGACAT-3′, MO-*zLpar3*: 5′-CAGCCCTAAAACAC AGGAAGACAT-3′. The MO stocks were prepared in sterile double-distilled water at a final concentration of 24 μg/ml. Each MO was injected at a 4-ng dose with 5% phenol red.

### O-dianisidine staining

O-dianisidine staining was used to study the expression of globin^49^. The embryos were collected at various stages of development and stained for 15 min in the dark with o-dianisidine (0.6 mg/ml), 0.01 M sodium acetate (pH 4.5), 0.65% H_2_0_2_, and 40% (V/V) ethanol. The stained embryos were cleared with benzyl benzoate/benzyl alcohol (2:1, V/V) and examined by differential interference contrast microscopy^50^.

### Whole-mount RNA *in situ* hybridization (WISH)

The embryos were digested with 10 μg/mL proteinase K and fixed with 4% paraformaldehyde. Next, the embryos were incubated with 0.015U of digoxigenin-labeled anti-sense RNA probes for 14–16 h at 60 °C. Anti-sense mRNA probes for *α-A1*, *β-E1*, *ikaros,* and *hbae1* were labeled with digoxigenin (Roche, Basel, Switzerland). The embryos were treated with RNase and washed to a stringency of 0.2× saline-sodium citrate buffer/hybridization buffer (SSC/HYB) at 60 °C. Finally, the embryos were exposed to Nitro tetrazolium blue/5-bromo-4-chloro-3-indoyl phosphate (Roche) in detection solution and incubated in the dark for 15–30 min^51^.

### Treatment of mice with LPA GPCR pharmacons

14-day-old male BALB/c mice (LASCO, Taipei, Taiwan) were dosed with a 0.5 mg/kg 2S-OMPT or 1 mg/kg GRI. Both chemicals were diluted in PBS with 3% BSA to a final volume of 300 μL and were injected peritoneally. Facial vein blood samples were taken on the seventh day and mixed with an equal volume of 2 mg/mL EDTA. The number of RBC, hemoglobin and hematocrit was analyzed using a Sysmex XT-2000i Automated Hematology Analyzer (Sysmex, Taiwan). All animal procedures were authorized and carried out in accordance with the approved guidelines and regulations of the Institutional Animal Care and Use Committee of the National Taiwan University.

### Statistical analysis

All the experiments were repeated at least three times. Significant differences were calculated using one-way analysis of variance. The statistical analyses were performed using StatView software (Abacus Concept). Results are expressed as the mean ± standard deviation based on at least three independent experiments. *p* < 0.05 was considered statistically significant in all the tests.

## Additional Information

**How to cite this article**: Lin, K.-H. *et al.* Pharmacological activation of lysophosphatidic acid receptors regulates erythropoiesis. *Sci. Rep.* **6**, 27050; doi: 10.1038/srep27050 (2016).

## Supplementary Material

Supplementary Information
