# Supplementary material for: Pharmacological activation of lysophosphatidic acid receptors regulates erythropoiesis
Source: Sci Rep. 2016 May 31;6:27050. doi: 10.1038/srep27050 (PMC4886675; doi:10.1038/srep27050)
Supplement: Supplementary Information [file srep27050-s1.pdf]

Pharmacological activation of lysophosphatidic acid receptors regulates erythropoiesis

Kuan-Hung Lin[1,†], Ya-Hsuan Ho[1,†], Jui-Chung Chiang[1], Meng-Wei Li[1], Shi-Hung Lin[1], Wei-Min Chen[1], Chi-Ling Chiang[2], Yu-Nung Lin[1], Ya-Jan Yang[3], Chiung-Nien Chen[4], Jenher Lu[5], Chang-Jen Huang[6], Gabor Tigyi[7], Chao-Ling Yao[8,*] and Hsinyu Lee[1,9,10,11,12,*]

[1]Department of Life Science, National Taiwan University, Taipei, Taiwan
[2]School of Biomedical Science, Ohio State University, Columbus, OH, USA,
[3]Graduate Institute of Oral Biology, School of Dentistry, National Taiwan University, Taipei, Taiwan
[4]Department of Surgery, National Taiwan University Hospital and College of Medicine, Taipei, Taiwan
[5]Department of Pediatrics and Pediatric Cardiology, Taipei Veterans General Hospital, National Yang Ming University, Taipei, Taiwan
[6]Department of Electrical Engineering, National Taiwan University, Taipei, Taiwan
[7]Department of Physiology, University of Tennessee Health Science Center, Memphis, TN, U.S.A.
[8]Department of Chemical Engineering and Materials Science, Yuan-Ze University, Taoyuan, Taiwan.
[9]Angiogenesis Research Center, National Taiwan University, Taipei, Taiwan
[10]Research Center for Developmental Biology and Regenerative Medicine, National Taiwan University, Taipei, Taiwan
[11]Center for Biotechnology, National Taiwan University, Taipei, Taiwan
[12]Institute of Biological Chemistry, Academia Sinica, Taipei, Taiwan

[†]**Authorship:** Kuan-Hung Lin and Ya-Hsuan Ho contributed equally to the work

[*]Co-Corresponding authors:

Dr. Hsinyu Lee

Address: No. 1, Sec. 4, Roosevelt Road, Taipei, Taiwan

Tel: +8862-3366-2499

Fax: +8862-2363-6837

E-mail: hsinyu@ntu.edu.tw

Dr. Chao-Ling Yao

Address: No. 135, Yuan-Tung road, Chung-Li District, Taoyuan City 32003, Taiwan

Tel: +886-3-4638800-3553

Fax: +886-3-4559373

E-mail: Yao@saturn.yzu.edu.tw

Supplemental Figures

Sup Fig 1. LPAR expression levels and knockdown efficiency

(a) The mRNA expression levels of LPAR1, LPAR2, and LPAR3 in K562 without hemin induction. (b) Hemin-induced γ-globin mRNA expression during 48 h. (c, d) Expression profiles of LPAR2 and LPAR3 during 48 h of hemin induction. The KD-efficiency of (e) LPAR2 by shRNA and (f) LPAR3 by siRNA. The quantitative data are represented as the mean ± SD based on at least three independent experiments. *$p < 0.05$ and **$p < 0.01$ indicate significant differences compared with vehicle control.

Sup Fig 2. The roles of LPA receptor in K562 cells under hemin induction.

(a-c) Knockdown of LPAR2 by lentiviral shRNA and (d-f) knockdown of LPAR3 by siRNA with hemin induction. CD71-FITC-conjugated and GlyA-PE-conjugated antibodies was used to stain and analyze the expression of CD71 and GlyA on the cell surfaces. The geometric mean of fluorescence was used to quantify the results. The quantitative data are represented as the mean ± SD based on at least three independent experiments. * $p < 0.05$, ** $p < 0.01$, indicate significant differences compared with vehicle control.

Sup Fig 3. LPAR expression profiling under LPAR agonist treatment

The mRNA expression levels of LPAR2 and LPAR3 were monitored by the treatments of (a, b) 5 μM and 10 μM of GRI977143, (c, d) 5 μM and 10 μM of DMP and (e, f) 80 nM and 100 nM of 2S-OMPT for 24 hr. The quantitative data are represented as the mean ± SD based on at least three independent experiments. *$p < 0.05$ indicate significant differences compared with vehicle control.

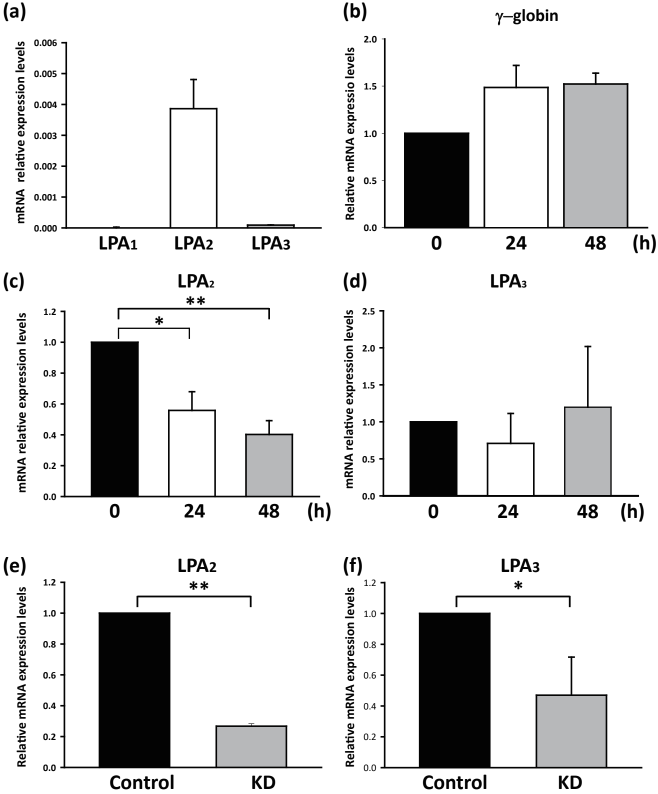
(a)
mRNA relative expression levels
0.006
0.005
0.004
0.003
0.002
0.001
0.000
LPA1
LPA2
LPA3
(b)
γ–globin
Relative mRNA expressio levels
2.0
1.5
1.0
0.5
0.0
0
24
48
(h)
(c)
LPA2
mRNA relative expression levels
1.2
1.0
0.8
0.6
0.4
0.2
0.0
**
*
0
24
48
(h)
(d)
LPA3
mRNA relative expression levels
2.5
2.0
1.5
1.0
0.5
0.0
0
24
48
(h)
(e)
LPA2
Relative mRNA expression levels
1.2
1.0
0.8
0.6
0.4
0.2
0.0
**
Control
KD
(f)
LPA3
Relative mRNA expression levels
1.2
1.0
0.8
0.6
0.4
0.2
0.0
*
Control
KD

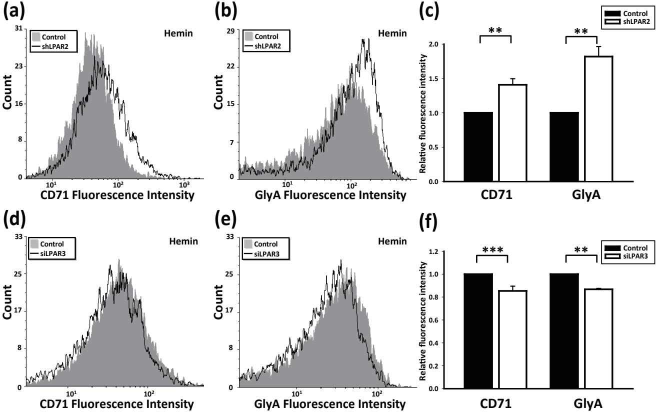

(a)
Control
shLPAR2
Hemin
Count
CD71 Fluorescence Intensity
(b)
Control
shLPAR2
Hemin
Count
GlyA Fluorescence Intensity
(c)
Control
shLPAR2
**
**
Relative fluorescence intensity
CD71
GlyA
(d)
Control
siLPAR3
Hemin
Count
CD71 Fluorescence Intensity
(e)
Control
siLPAR3
Hemin
Count
GlyA Fluorescence Intensity
(f)
Control
siLPAR3
***
**
Relative fluorescence intensity
CD71
GlyA

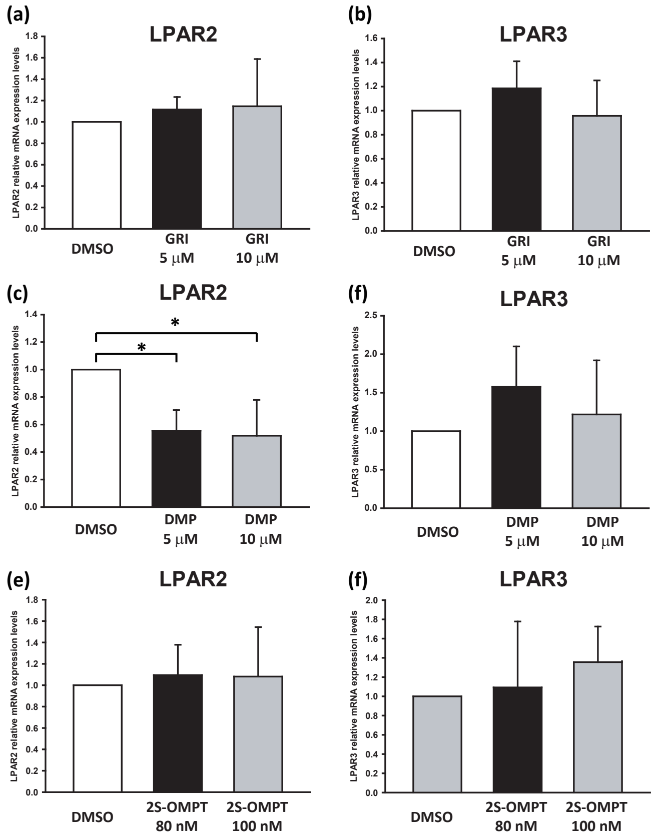
(a)
LPAR2
LPAR2 relative mRNA expression levels
0.0
0.2
0.4
0.6
0.8
1.0
1.2
1.4
1.6
1.8
DMSO
GRI 5 μM
GRI 10 μM
(b)
LPAR3
LPAR3 relative mRNA expression levels
0.0
0.2
0.4
0.6
0.8
1.0
1.2
1.4
1.6
DMSO
GRI 5 μM
GRI 10 μM
(c)
LPAR2
LPAR2 relative mRNA expression levels
0.0
0.2
0.4
0.6
0.8
1.0
1.2
1.4
*
*
DMSO
DMP 5 μM
DMP 10 μM
(f)
LPAR3
LPAR3 relative mRNA expression levels
0.0
0.5
1.0
1.5
2.0
2.5
DMSO
DMP 5 μM
DMP 10 μM
(e)
LPAR2
LPAR2 relative mRNA expression levels
0.0
0.2
0.4
0.6
0.8
1.0
1.2
1.4
1.6
1.8
DMSO
2S-OMPT 80 nM
2S-OMPT 100 nM
(f)
LPAR3
LPAR3 relative mRNA expression levels
0.0
0.2
0.4
0.6
0.8
1.0
1.2
1.4
1.6
1.8
2.0
DMSO
2S-OMPT 80 nM
2S-OMPT 100 nM
